# Human Flicker Fusion Correlates With Physiological Measures of Magnocellular Neural Efficiency

**DOI:** 10.3389/fnhum.2018.00176

**Published:** 2018-05-14

**Authors:** Alyse Brown, Molly Corner, David P. Crewther, Sheila G. Crewther

**Affiliations:** ^1^School of Psychological Science and Public Health, La Trobe University, Melbourne, VIC, Australia; ^2^Centre for Human Psychopharmacology, Swinburne University of Technology, Melbourne, VIC, Australia

**Keywords:** magnocellular, neural efficiency, visual evoked potential (VEP), flicker fusion, non-linearities

## Abstract

The rapidity with which the visual system can recover from stimulation in order to respond again has important implications for efficiently processing environmental stimuli in real time. To date, there has been little integration of the human psychophysical and physiological research underlying the neural mechanisms contributing to temporal limits on human visual perception. Hence, we investigated the relationship between achromatic flicker fusion frequency and temporal analysis of the magnocellular (M) and parvocellular (P) contributions to the achromatic non-linear multifocal Visual Evoked Potential (mfVEP) responses recorded from occipital scalp (Oz). It was hypothesized, on the basis of higher temporal cut-off frequencies reported for primate M vs. P neurons, that sinusoidal flicker fusion frequencies would negatively correlate with the amplitude of M- but not P-generated non-linearities of the mfVEP. This hypothesis was borne out in 72 typically developing young adults using a four-way forced choice sinusoidal flicker fusion task: amplitudes of all non-linearities that demonstrated a clear M-generated component correlated negatively with flicker thresholds. The strongest of these correlations were demonstrated by the main M non-linearity component (K2.1_N70−P100_) for both high contrast (*r* = −0.415, *n* = 64, *p* < 0.0005) and low contrast (*r* = −0.345 *n* = 63, *p* < 0.002) conditions, indicating that higher achromatic flicker fusion threshold is linked to a more efficient (smaller second order kernels) M system. None of the peaks related to P activity showed significant correlations. These results establish flicker thresholds as a functional correlate of M-pathway function as can be observed in the non-linear analysis of mfVEP.

## Introduction

The speed with which the brain processes visual information has important implications for our ability to attend, process, and respond to environmental stimuli. Logic also suggests that the temporal processing capacity and sensitivity of the Magnocellular (M) and Parvocellular (P) subcortical pathways must depend in part on the rapidity of neural recovery after visual stimulation. In line with this reasoning, flicker fusion thresholds for rapidly modulated luminance (Hecht and Shlaer, 1936; de Lange Dzn, 1954) have long been used clinically as a measure of rates of perceptual processing (Brenton et al., 1989).

Flicker fusion thresholds are known to change for an individual as a function of viewing distance, stimulus size, intensity, luminance, achromatic vs. chromatic, and retinal location (Brenton et al., 1989). Achromatic flicker fusion thresholds are reported to be in the range of 35 to 60 Hz (Hecht and Shlaer, 1936; de Lange Dzn, 1954), depending on flicker modulation depth, while color fusion from red/green isoluminant flicker occurs at much lower frequencies (10–15 Hz Wisowaty, 1981; Schiller et al., 1991). This difference in chromatic and achromatic flicker fusion rate has typically been attributed to the different pathways that control the threshold frequency, i.e., either the slow sustained, red/green sensitive P pathway or the fast transient achromatic M pathway (Derrington and Lennie, 1984; Derrington et al., 1984). Temporal structure of cortical visual evoked responses has previously been probed physiologically in humans (Klistorner et al., 1997) using Wiener kernel analysis of pseudo-randomly modulated luminance patches. Klistorner et al. (1997) demonstrated that the first slice of the second order kernel (K2.1) showed high contrast gain at low contrast with response saturation at higher contrast, while the second slice (K2.2) showed lower contrast gain and no saturation of peak response amplitudes. The second slice K2.2 also showed a peak latency ~25 ms later than that of the K2.1 (Klistorner et al., 1997; Sutherland and Crewther, 2010; Jackson et al., 2013). These temporally separable major contributions to the first and second slices of the second order kernel (K2.1 and K2.2, respectively) were identified as generated by M and P pathways respectively, on the basis of similarity with the achromatic contrast response functions of M and P cells recorded in primate lateral geniculate nucleus (Derrington and Lennie, 1984; Kaplan and Shapley, 1986; Kaplan et al., 1987; Lee et al., 1990). This idea that M and P contributions to cortical processing can be independently identified is not universally accepted. Skottun (2013) has suggested that the M signal cannot be isolated by high temporal frequencies as filtering occurs in primary visual cortex with temporal cut-offs in cortical neurons that are ~20 Hz lower than in monkey LGN cells (Hawken et al., 1996), with recent human findings in agreement (Bayram et al., 2016). However, we would argue that Wiener kernel analysis of the VEP precisely captures such cortical filtering, and through differences in temporal filtering, contrast responses, and evoked latencies between different cell classes, can extract separate contributions.

The contribution of the third cell type in LGN the konicellular (K) class to the mfVEP should be considered. Originally found within the interlaminar regions of the lateral geniculate (K1-K6), they have now been isolated also within M and P cellular layers of the LGN (Hendry and Reid, 2000). Hence, though K cells are just as numerous as M cells, they are heterogeneous in response resulting in a further diminishing contribution to the average evoked response. Furthermore, K cells have slow input latencies to cortex (10 ms longer than P) (Pietersen et al., 2014) and do not appear to contribute an observably unique contribution to an achromatic mfVEP.

Wiener kernel analysis has recently been applied to show group differences in M and P function for populations selected across the autistic spectrum (Sutherland and Crewther, 2010; Jackson et al., 2013; Brown and Crewther, 2017) as well as in a group of neurotypical young adults following an omega-3 fatty acid dietary supplementation (Bauer et al., 2011). Bauer et al. (2011), using a cross-over design demonstrated smaller amplitude second order M-non-linearities and shorter choice reaction times for the omega-3 oil EPA (eicosapentaenoic acid) compared with No Diet conditions, and suggested that an EPA enriched diet induced faster neural recovery for the M-pathway.

In order to account for individual variation in evoked potentials, a scaling of the M and P generated peaks by the corresponding peaks of the first order response is sensible. Thus, Brown and Crewther (2017) used the amplitude ratios K1_N70−P100_/K2.1_N70−P100_ and K1_N140−P180_/K2.2_N110−P150_ as measures of M and P neural efficiencies in comparing the VEP from an autism spectrum disorder (ASD) child population compared with a typically developing control group.

To date, there is no general population study correlating psychophysically measured rates of information processing and M and P neural efficiencies derived from human non-linear mfVEP.

Thus, the aim of the current study was to compare psychophysically determined flicker fusion frequencies with Wiener kernel derived M and P contributions to the non-linearities generated in electrophysiological recordings over primary visual cortex, for conditions of high and low temporal contrast (modulation). On the basis of M cells in primate LGN demonstrating higher temporal cutoff frequencies, it was predicted that flicker fusion frequencies would correlate negatively with magnocellular but not with parvocellular generated VEP non-linearities.

## Methods

### Participants

Following approval from the Latrobe University Human Research Ethics Committee, 81 first year psychology students were recruited for the study. Data was collected from 71 females (age in years: *M* = 21.14, *SD* = 5.01) and 10 males (age in years: *M* = 20.60, *SD* = 2.63), the differences in ages being insignificant. On arrival at the laboratory, informed consent was obtained from participants and all participants were carefully screened for a history of epilepsy; none were excluded.

### Flicker fusion

Achromatic flicker fusion frequencies were measured at high (75%) and low contrast (5%) using separate experimental runs. The LEDs (A-Bright Industrial Co., China, part AL-513W3c-003 white) were controlled by VPixx software driving the four analog outputs of a DATAPixx interface device (www.vpixx.com). With sampling at 10 kHz, this allows for a smooth sinusoidal waveforms with frequencies in excess of 100 Hz. The light from the four LEDs was conveyed into separate optic fiber light guides (6 mm diameter), terminating flush with a drilled wooden display panel, where the 4 light guides were arranged in a diamond shaped array, giving a mnemonic top, bottom, left, and right concordance with the button box employed. Viewed from 60 cm, the separation of the light guides subtended 1.0°, center-to-center, at the eye. A ColorCal II colorimeter was used to calibrate and linearise the luminance of each LED (MkII, Cambridge Research Systems) with maximum luminance adjusted to 86 cd/m^2^. In order to prevent the alerting of change sensitive mechanisms, a gaussian temporal envelope (FWHM = 480 ms) was used to smooth the onset and offset of the flicker. A four-way forced choice design was employed consisting of 32 trials. A Parameter Estimation by Sequential testing (PEST) procedure, embedded in VPixx was used to establish flicker fusion thresholds for both high and low temporal modulation conditions. The order of High and Low contrast conditions was counterbalanced in order to control for practice effects.

Participants completed the tasks in a light controlled, dimly illuminated lab enviroment. Each trial ran for 3 s, beginning with a high pitch beep to indicate the start of testing and finished with a low pitch beep to mark the end of the trial. Participants were informed that one target light would flicker each trial, and were instructed that at the end of the trial they were to use the button box in front of them to manually indicate which of the four lights flickered. When the participant was unsure of the answer they were required to guess which light they thought was the target. Participants were given an initial practice session containing 10 trials which covered flicker frequencies into the threshold regions and back again to perceivable flicker conditions to familiarize participants with all aspects of the task.

### mfVEP

Participants completed two electrophysiological recordings, each of 4-min duration. Participants were seated 70 cm away from the screen and instructed to look at a central red dot while recordings took place. Non-linear mfVEPs were recorded from the primary visual area (V1) located in occipital cortex. Using the 10/20 system, gold plated electrodes were placed over the recording site Oz, with Fz used as the reference and the right mastoid as ground. The stimulus was created and run in VPixx and employed a DATAPixx (www.vpixx.com) interface box for strict video frame registration. The stimulus was presented on a 75 Hz frame rate CRT 21″ monitor (ViewSonic E90). The stimulus array was kept at a constant mean luminance of 52 cd/m^2^ as measured by a ColorCal (MkII, Cambridge Research Systems) probe.

**Figure 1:**
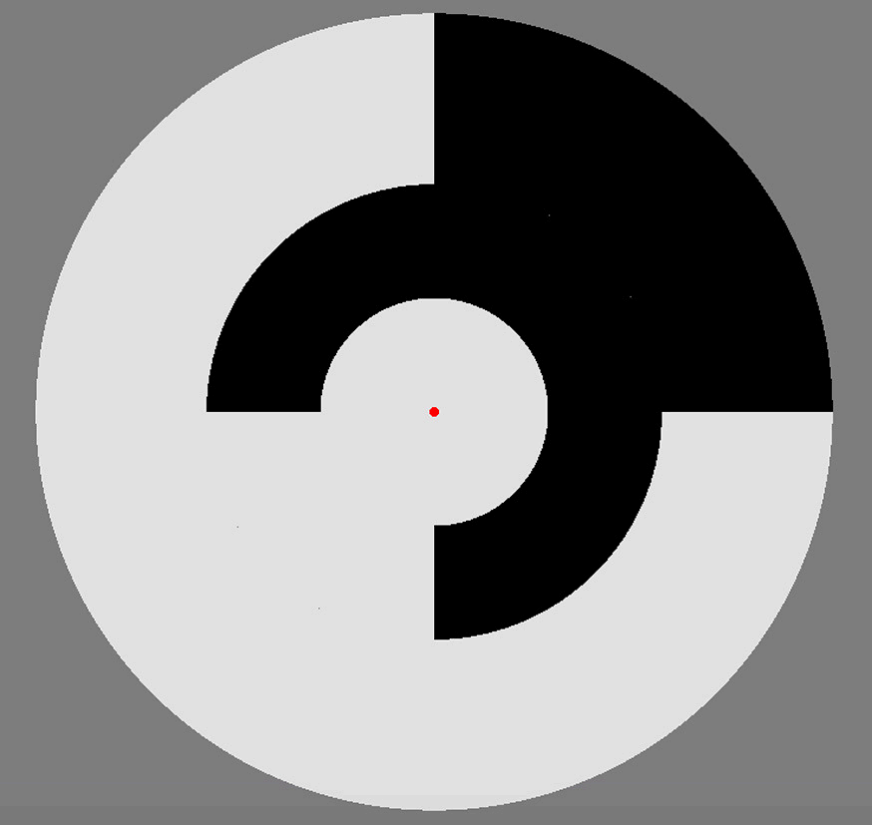
Multifocal stimulus employed for the VEP recordings. A central 7° circular disk was surrounded by two annuli each divided into four separate patches. Each patch fluctuated between two luminance levels on the basis of a pseudorandom m-sequence, with the sequence for each patch maximally shifted, resulting in stimuli that are mutually decorrelated.

A circular multifocal stimulus (see Figure 1) containing nine segments (two outer rings each divided into four patches plus a central patch) was employed, with a red dot central fixation point. Luminance levels of each patch fluctuated using a pseudo-random binary multifocal m-sequence. The stimuli were presented as achromatic unstructured segments hence the patches are diffuse defined only by events where they differ in contrast luminance. The m-sequence allows for an equal occurrence of different binary patterns during the sequence which results in a similar number of response events for each Wiener kernel analysis (see data pre-processing individual sequence analysis). Two different contrast presentation conditions 24 and 96% temporal contrast as recommended by Klistorner et al. (1997), for maximum separability of M and P contributions. The 4 min binary m-sequence was broken into 4 one min segments to prevent fatigue and to allow for eye blinks.

### Data pre-processing

Only responses from the central patch of the stimuli (subtending 7° of visual angle) were analyzed with the peripheral patches employed to eliminate edge contributions to the evoked responses. Curry 7 (compumedicsneuroscan.com) recording software was used to collect data. The signal was amplified 10,000 times, notch filtered at 50 Hz, and was sampled at a rate of 1 kHz. Eye blinks were removed manually and a base line correction (removing the averages from −50 to 0 ms). At this stage of analysis nine participants were removed from the study due to the poor quality of the recording leaving data from 72 individuals to be analyzed. Curry received a series of triggers from VPixx each frame (13.33 ms), from which epochs 100 ms pre- and 500 ms post-trigger were collected. For a binary White/Black sequence, the first order response (K1) corresponds to one half of the average of all responses to a white stimulus minus the average of all responses to a black stimulus 0.5^*^(R_W_-R_B_). The second-order, first and second slice, responses (K2.1, K2.2) measures the effect of prior stimulation on the response of the current stimulation frame. K2.1 represents a comparison between two consecutive frames when a transition has occurred to when a transition has not: K2 = 0.25^*^ (R_BB_ + R_WW_ - R_BW_ + R_WB_). K2.2 also measures the effect of preceding stimulation but with an additional intervening frame of either polarity.

Amplitudes and latencies of peaks from the VEP recordings were extracted using IGOR Pro (Wavemetrics, USA). Waves were then interpreted as being generated by magnocellular or parvocellular afferents with criteria of contrast gain, saturation at high contrast, and latency differences between components provided by previous research (Baseler and Sutter, 1997; Klistorner et al., 1997; Crewther et al., 1999; Laycock et al., 2007; Sutherland and Crewther, 2010; Jackson et al., 2013).

Mean average waves of the 72 participants were calculated for each kernel and latency and peak to peak amplitude data was correlated with the flicker fusion. One-tailed Pearson correlations were performed to examine the relationship between flicker fusion and non-linear VEP group amplitude and latency data. Age of participants was controlled in the analyses. To correct for the multiple comparisons being made, an alpha value of 0.005 was used. Mahalanobis distances were used to detect outliers. To reduce between-subject variation in recording conditions (e.g., skull thickness and muscle artifact), ratios of the second order to first order amplitudes of the prominent M and P generated peaks were calculated for each participant. The M and P efficiency are defined here as the first order to second order amplitude ratios. Thus, M-efficiency = K1_N70−P100_/K2.1_N70−P100_; P-efficiency = K1_N140−P180_/K2.2_N110−P150_.

## Results

### Flicker fusion

Behavioral performance on the flicker fusion tasks showed an effect of contrast with the 5% modulation showing a lower mean threshold than the 75% modulation (see Table 1). This result is consistent with the literature on flicker fusion contrast function (Thompson et al., 2015). A paired samples *t*-test revealed a significant difference in flicker fusion between 5 and 75% contrast stimuli *t*_(74)_ = 9.37, *p* < 0.0005.

**Table 1:** Flicker fusion.

	**Mean (Hz)**	***SD***	***n***
75% modulation	47.82	3.45	76
5% modulation	44.10	3.26	78

### Non-linear VEP

**Figure 2:**
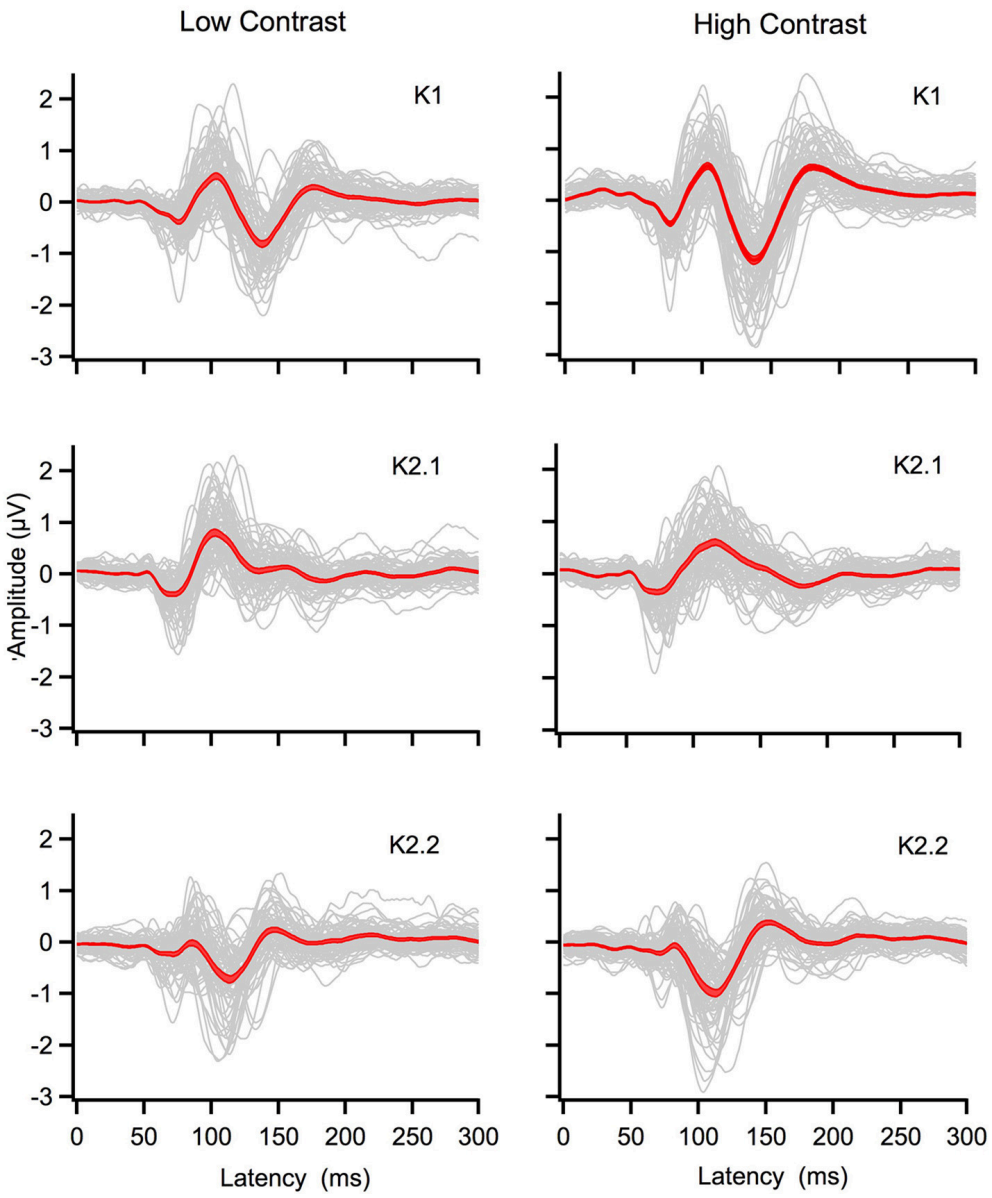
Individual (gray) and grand mean averages with standard errors (red) for the first and second order kernel responses generated by the central patch of the multifocal stimulus. The left column shows K1, K2.1, K2.2 responses for low contrast (24%) while the right column shows responses for high contrast (96%).

Grand mean averages of the K1, K2.1, and K2.2 kernels for the low and high temporal contrast recordings are displayed in Figure 2. Main peak amplitudes are located around the following latencies: high contrast K1: N80, P100, N140, P180; K2.1: N70, P110, N140; K2.2: N70, P80, N110, P150, and at low contrast K1: N70, P100, N140, P170; K2.1: N70, P100, N140; K2.2: N70, P90, N110, P150. Inspection of the graphs shows that the K1 and K2.2 waveforms increase in amplitude as a function of contrast, while the amplitude of the main K2.1 kernel response does not increase—evidence that response saturation has already occurred by 24% contrast level.

### Correlations: flicker frequency cf non-linear VEP

**Figure 3:**
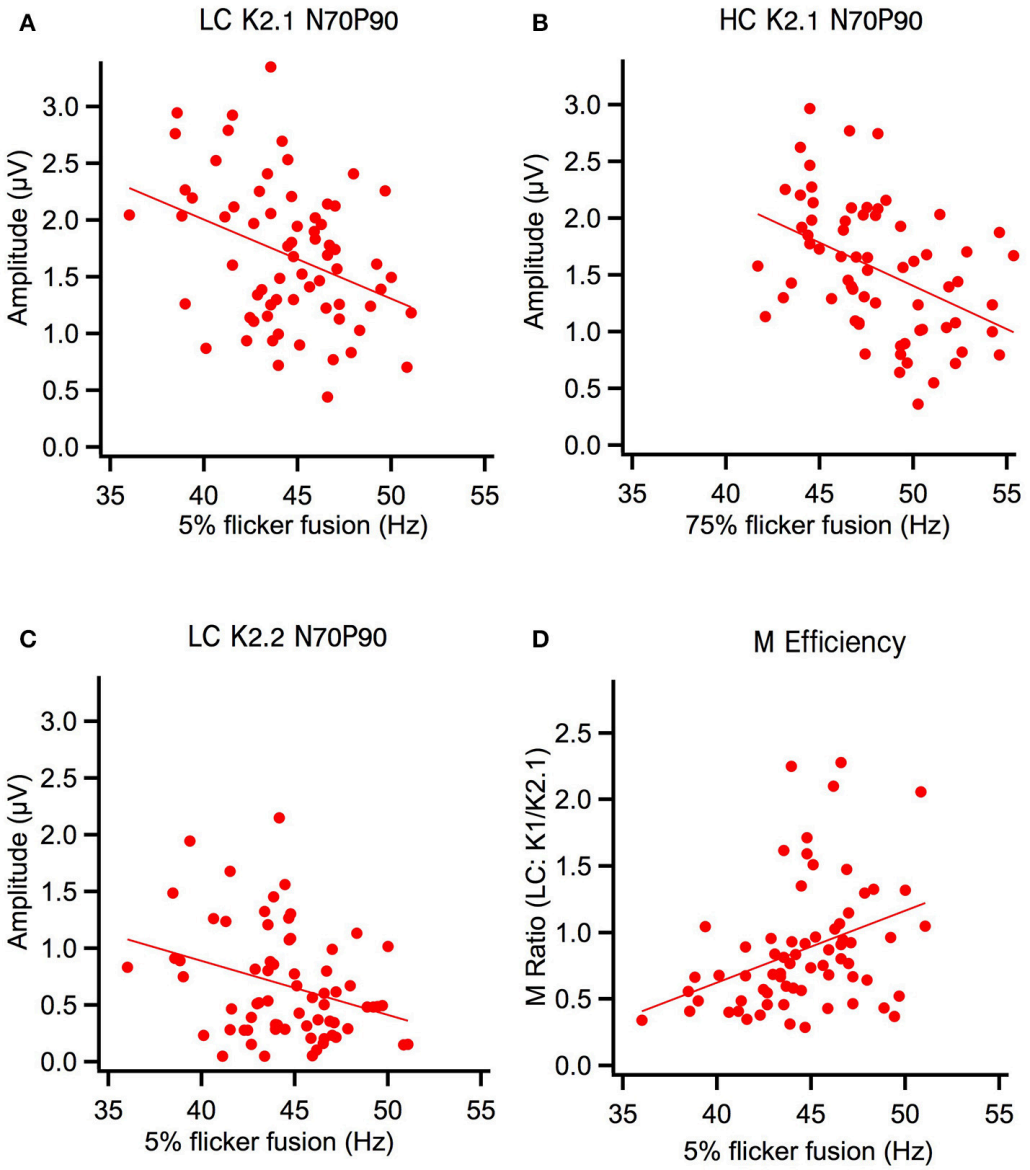
Flicker fusion at high and low contrast (HC, LC) correlated negatively with early second order amplitude components and positively with the M ratio. **(A)** Low contrast K2.1_N70−P100_ amplitude component was significantly correlated with low contrast flicker and **(B)** high contrast K2.1_N70−P100_ amplitude component was significantly correlated with high contrast flicker. **(C)** At low contrast the K2.2_N70−P100_ amplitude component was also significantly correlated with low contrast flicker. **(D)** Magnocellular efficiency increases as a function of low contrast flicker fusion frequency.

One-tailed Pearson correlation tests showed that peak-to-peak amplitude and flicker thresholds in the first order kernels were not correlated. However, amplitudes of the major waveform of the first slice of the second order kernel (N70-P100) were negatively correlated with flicker fusion (see Table 2). Irrespective of contrast conditions, these K2.1 peak-to-peak amplitudes correlated negatively with flicker fusion frequencies. The correlation between the high contrast conditions (K2.1_N70−P110_ with 75% flicker) explained most variance in the correlation (17%) with the correlation between the low contrast conditions (K2.1_N70−P100_ with 5% flicker) explaining a lesser amount (11%). In K2.2_N70−P90_ only the low contrast peak-to-peak amplitude correlated with the flicker thresholds while the high contrast K2.2_N70−P80_ did not. Analysis of peak latencies revealed a correlation between the 5% flicker threshold and the N70 latency (*r* = −0.318, *p* < 0.005) and P100 latency (*r* = −0.369, *p* < 0.001) of the short latency (M-generated) K2.2 peaks at low contrast, with faster fusion frequencies associated with shorter latencies (see Figure 3). No further correlations were found between peak latencies and flicker fusion. Independent *t*-tests showed that gender did not influence the data.

**Table 2:** Pearson *r* and significance level for correlations between low contrast flicker fusion and the low contrast peak-to-peak amplitudes and the M efficiency ratio of the VEP, as well as correlations between high contrast flicker fusion and the high contrast peak to peak amplitudes and P efficiency ratio of the VEP.

**VEP**	**Flicker fusion**	
	**Low contrast *df*(64)**	**High contrast *df*(63)**
First order kernel LC K1 (N70-P100)	*r* = 0.075, *p* = 0.274	
LC K1 (N140-P170)	*r* = −0.052, *p* = 0.338	
HC K1 (N80-P100)		*r* = −0.181, *p* = 0.074
HC K1 (N140-P180)		*r* = −0.116, *p* = 0.159
Second order kernel slice 1 LC K21 (N70-P100)	*r* = −0.345^*^, *p* < 0.002	
HC K21 (N70-P110)		*r* = −0.415^**^, *p* < 0.000
Second order kernel slice 2 LC K22 (N70-P80)	*r* = −0.320^*^, *p* < 0.004	
LC K22 (N110-P150)	*r* = −0.041, *p* = 0.456	
HC K22 (N70-P90)		*r* = −0.213, *p* = 0.044
HC K22 (N110-P150)		*r* = −0.016, *p* = 0.450
LC M efficiency	*r* = 0.314^*^, *p* < 0.005	
HC P efficiency		*r* = −0.132, *p* = 0.147

p < 0.005^*^, p < 0.0005^**^, LC, low contrast; HC, high contrast.

*correlational analysis of the flicker thresholds with amplitudes were analyzed in separate one tailed Pearson list-wise tests, corrected for multiple comparisons*.

## Discussion

This study, with a large non-selected population of young adults shows a clear relationship between the amplitude in the second order non-linear mfVEP (K2.1_N70−P100_) responses that have previously been associated with M pathway activation by Klistorner et al. (1997) and Jackson et al. (2013). As predicted, we have shown that higher flicker fusion correlates with peak-to-peak amplitude reduction in the early K2.1 component of the mfVEP regardless of contrast condition. A similar correlation is also present in the early amplitude of the low contrast second slice non-linearity (K2.2_N70−P80_) component where M input still produces a clear N70 negativity (Klistorner et al., 1997; Sutherland and Crewther, 2010; Bauer et al., 2011; Jackson et al., 2013; Brown and Crewther, 2017). Interestingly, Klistorner et al. (1997) did not record this K2.2 contribution of the M system, possibly because their mfVEP was carried out with a frame rate of 67 Hz rather than 75 Hz. As recorded here, the K2.2 early contribution is only one-sixth of the K2.1 amplitude, and with a capacity of around 50 Hz flicker fusion, one would expect recovery in about 20–30 ms, a figure that fits nicely with the two frames of the second order response (either 30 or 26 ms depending on the frame rate). However, at high contrast the K2.2 N70 component is not present in all participant recordings (see Figure 2), presumably being subsumed by the much larger, longer latency P-generated peak. This could explain why the high contrast K2.2 response was not correlated with flicker fusion as seen in the K2.2 response at low contrast here. These data present a strong case for M response influencing perceptual speed of visual processing. In addition, our methods can measure this function non-invasively in humans. Importantly, flicker fusion remains correlated when individual ratios of participants' prominent M generated peaks (K1_N70−P100_ / K2.1_N70−P90_) were made to reduce between-subject variation in the recording. Using this ratio, better M efficiency (larger K1 to K2.1 amplitudes) correlated with higher flicker fusion at low contrast. This study, examining visually evoked non-linearities and perceptual processing speed (flicker fusion) is the first to directly correlate flicker fusion behavior and VEP physiological components.

The strongest of the correlations was found between the high contrast conditions of both tasks: 75% temporal modulation flicker frequency and 96% contrast multifocal stimulus. This difference is presumably due to an improvement in signal to noise at high contrast. Importantly, there were no additional significant correlations between flicker thresholds and the first order responses or the later peak-to-peak components from N140 to P180 of the non-linearities (see Table 2). Hence, there was no flicker fusion correlations associated with first order responses or responses identified as of parvocellular source.

Adhering to the contrast response function of the M system, the flicker fusion for the current study showed the expected threshold increase from 5% contrast to 75% contrast (Brenton et al., 1989). However, in comparison with a previous study by Thompson et al. (2015) who used the same flicker apparatus, the current study found considerably higher thresholds for the 5% contrast condition. It is possible that procedural differences in presentation of contrast conditions between the two studies altered the 5% contrast threshold. Here, the two contrast conditions were thresholded separately, whereas Thompson randomly interleaved 5 contrast conditions (ranging from 5 to 100%) within the one task, where saliency of the 5% flicker may have been reduced due to high contrast adaptation, hence resulting in a lower mean flicker fusion.

Variation of differences in amplitude and latency between groups have been identified across non-linear VEP studies (Sutherland and Crewther, 2010; Bauer et al., 2011; Jackson et al., 2013; Brown and Crewther, 2017). Between these studies, changes in the amplitude of the main peak-to-peak M and P non-linear components (K2.1_N70−P100_; K2.2_N110−P150_) are found with remarkable consistency. This current study helps link the theory of measurable neural recovery in recorded non-linearities to individual differences in the amplitude of all non-linearities that have a clear M generated N70 component.

## Author contributions

AB was the primary contributor to this study and was involved in the design, research theory, data collection, and write up; MC was an honors student who helped with the data collection; DC and SC co-supervised this study and were involved in the design and the development of the theory and helped with the write up of the manuscript.

### Conflict of interest statement

The authors declare that the research was conducted in the absence of any commercial or financial relationships that could be construed as a potential conflict of interest.
